# All or nothing? Partial business shutdowns and COVID-19 fatality growth

**DOI:** 10.1371/journal.pone.0262925

**Published:** 2022-02-09

**Authors:** Matthew Spiegel, Heather Tookes

**Affiliations:** Yale School of Management, Yale University, New Haven, Connecticut, United States of America; Istanbul Medeniyet University: Istanbul Medeniyet Universitesi, TURKEY

## Abstract

Incomplete vaccine uptake and limited vaccine availability for some segments of the population could lead policymakers to consider re-imposing restrictions to help reduce fatalities. Early in the pandemic, full business shutdowns were commonplace. Given this response, much of the literature on policy effectiveness has focused on full closures and their impact. But were complete closures necessary? Using a hand-collected database of partial business closures for all U.S. counties from March through December 2020, we examine the impact of capacity restrictions on COVID-19 fatality growth. For the restaurant and bar sector, we find that several combinations of partial capacity restrictions are as effective as full shutdowns. For example, point estimates indicate that, for the average county, limiting restaurants and bars to 25% of capacity reduces the fatality growth rate six weeks ahead by approximately 43%, while completely closing them reduces fatality growth by about 16%. The evidence is more mixed for the other sectors that we study. We find that full gym closures reduce the COVID-19 fatality growth rate, while partial closures may be counterproductive relative to leaving capacity unrestricted. Retail closures are ineffective, but 50% capacity limits reduce fatality growth. We find that restricting salons, other personal services and movie theaters is either ineffective or counterproductive.

## Introduction

Beginning in March 2020, government entities began to respond to the COVID-19 crisis by shutting down businesses and many social activities. The early business restrictions were simple; establishments were open or closed. Consequently, the early literature focused on full closures and their impact [1–9]. Once cases started to decline, governments often responded by letting businesses partially reopen. The hope was to mitigate economic costs while keeping COVID-19 under control. How effective were partial shutdowns compared to full closures? This question is of considerable interest since incomplete vaccine rollouts and limited vaccine availability to some segments of the population (e.g., children) may lead policymakers to consider imposing new restrictions to help reduce fatalities. This paper uses hand-collected county-level data to analyze the impact of partial shutdowns of restaurants, bars, gyms, spas, retail establishments, and movie theaters on the growth of COVID-19 fatalities. (We use the term “spas” to refer to all barbershops, salons, and other personal services.) The data that we use offer far greater granularity relative to datasets used by others studying the effectiveness of non-pharmaceutical policy interventions (NPIs) on COVID-19 deaths in the U.S.

We begin with the database used in [8]. It contains U.S. county-level policy restrictions for the period March 1, 2020 through December 31, 2020. The database captures full closures and lockdowns, including: general business closures; specific closures targeting bars, restaurants, gyms, and spas; no visitation policies at nursing homes; mandatory mask orders; park and beach closures; and limits on the size of gatherings. Because many policies have also targeted retail establishments and movie theaters, we add those sectors to the database. In total, we track six specific sectors: restaurants, bars, gyms, spas, retail establishments, and movie theaters. These are the businesses for which capacity restrictions are common and where understanding whether it is possible to limit the spread of COVID-19 while keeping businesses at least partially open is of particular interest. To examine the impact of partial closures, we introduce a range of sector-specific capacity limits. For each county or state government mandate, we record a restriction level and a start date. Table 1 contains a list of key terms that describe the restrictions that we analyze. Data access information can be found in S1 Methods.

**Table 1:** Restaurant, bar, gym, spa, retail, and movies restrictions.

Keyword	Description
Closed	For restaurants and bars, closed or limited to takeout. For gyms, spas, retail establishments, and movie theaters, closed or limited to servicing customers outdoors.
Out	For restaurants and bars, limited to outdoor service. No indoor service permitted.
25%	Facility open, but under an indoor capacity limit of between 1% and 25% of indoor capacity.
50%	Facility open under an indoor capacity limit greater than 25% and less than or equal to 50%.
>50%	Facility open with indoor capacity over 50%, up to and including 100%.
Restaurants X	Restaurants under capacity restriction X (where X is Close, Out, 25%, 50% or >50%)
Bars X, Rest Y	Bar under capacity restriction X (where X is Close, Out, 25%, 50% or >50%) and restaurants are simultaneously under capacity restriction Y (where Y is Close, Out, 25%, 50% or >50%).
Gyms X	Gyms under capacity restriction X (where X is Close, 25%, 50% or >50%)
Spas X	Spas under capacity restriction X (where X is Close, 25%, 50% or >50%)
Retail X	Retail under capacity restriction X (where X is Close, 25%, 50% or >50%)
Movies X	Movies under capacity restriction X (where X is Close, 25%, 50% or >50%)

As Table 1 indicates, we do not track outdoor regulations for gyms, spas, retail establishments, and movie theaters. In the rare instances in which these businesses are restricted to outdoor activities, we classify them as closed. During our sample period, restrictions on bars are always as strict as or stricter than those on restaurants. As such, we can only study the impact of restricting bar capacity, given a specific restriction on restaurants. To account for this, we focus the analysis on restaurant-bar restriction pairs (Bars X, Rest Y). The S1 Table shows summary statistics for the policy variables on which we focus the analysis. The S2 Table Panel A summarizes the number of weeks each policy remains in force.

## Empirical analysis

Our regressions forecast the week *t*+*k* rate of fatality growth based on data as of period *t*:

$$100G_{c,}{}_{t+k}=\alpha +\sum_{p}\beta_{p,k}D_{c,p,t}+controls+\varepsilon_{c,t+k}$$ (1)

In Eq (1) *p* is an indicator for the policy restriction put into place in county *c* at date *t*. The β are estimated coefficients and *D* are policy dummies. Dummies equal 1 if a particular policy is in place at time *t* and are zero otherwise. If multiple restrictions are imposed during a particular week, the restriction covering the most days is set to 1 and the others to 0. For gyms, spas, retail establishments, and movie theaters, capacity limits of over 50% are the omitted variable. For restaurants and bars, the omitted variable is one indicating that both can open beyond 50% of capacity. Standard errors are clustered at the county level and are robust to heteroskedasticity

Interpreting the coefficient estimates from Eq (1) is straightforward. We hold constant an area’s demographics, weather, past COVID-19 fatality growth and other policies in place as of date *t*. The β_*p*,*k*_ coefficients then imply that, *k* weeks after policy *p* is in place, the county’s fatality growth rate will, on average, differ by β_*p*,*k*_ relative to an identical county without the policy in place.

Potential false positives and false negatives are natural concerns when evaluating policy data. Suppose government entities introduce rules near the natural peak of fatality growth.^1^ In this case, ineffective or even somewhat counterproductive restrictions can appear to be beneficial–a false positive. False negatives are also possible when effective policies are introduced as fatalities are accelerating. To combat these twin endogeneity problems, we take four approaches.

First, estimates of Eq (1) include a wide array of controls. These include county demographics, weather, and a variety of non-business restrictions, such as limits on gathering sizes, park closures, and mask policies. Earlier studies of NPIs during the COVID-19 pandemic have shown that these variables are closely associated with COVID-19’s spread [3–7, 10–13] and result in reduced population movement.[14–16] Similar to [8], we analyze counties rather than states (or countries). While many orders originate at the state level, they often apply to just a subset of counties. Conversely, some counties instituted policies apart from those issued by the state. County data more accurately reflect the policies in place and provide considerable statistical power relative to state our country level data others have used.[10, 11, 13, 14]

Every test controls for the total number of fatalities to date, the time since the first reported fatality in the county, the time since March 1, 2020, and six lags of weekly fatality growth. These help identify which policies alter the trajectory of transmission and death versus those that happen to be implemented during its natural rise or fall within a community.

Our second strategy for mitigating the endogeneity issue uses the trend in fatalities at the time a policy is enacted. We only consider a policy effective or counterproductive if the trends in fatality growth at the time of policy introduction (i.e., during the first and second week after a policy’s enactment) are either both insignificantly different from zero or, if significant, have a sign opposite that of the forecast horizon effect.

Third, we try to identify potential false positives by removing each state’s most populous counties and then repeating the regression analysis. Presumably, state governments focus their attention on more populated areas. When counties fall under orders optimized for other more populous parts of the state, we can estimate the impact of “out-of-equilibrium” policies on future fatalities. This should help identify false positives (e.g., policy introductions at the county’s natural fatality peak).

Fourth, we conduct “near-border” tests that are similar to the standard nearest neighbor analysis to further mitigate endogeneity problems. Instead of examining matched pairs of counties that lie on state borders (as a traditional nearest neighbor pairing would), we require at least a one-county buffer between a near-border county and a state line. Given the way that COVID-19 spreads, a policy that affects transmission in one county is likely to have a direct impact on an adjacent one. For example, different regulations for dining in between Queens County and Nassau County in New York caused some patrons to dine in Nassau County [17]. The same happened when Georgia opened its restaurants and its neighboring states did not. Similarly, Pennsylvania residents traveled to Ohio to purchase alcohol when the two states had differing restrictions [18]. Researchers, using more formal tests, have also found that stay-at-home orders in one area affected movement in others [6]. The geographic buffer in the near-border tests help address this issue while also using geographic proximity to control for the possibility that policies simply reflect local trends in the trajectory of the virus.

## Results and discussion

The tables that follow include results of estimating Eq (1) when *k* is set to 4 and 6, corresponding to the growth rate in fatalities *k* weeks after a policy is put in place. The CDC reports a median incubation period from exposure to symptom onset of 4–5 days. Among people with severe disease, the median time to ICU admission from the onset of illness or symptoms ranges from 10–12 days [15]. For patients admitted to the hospital and who do not survive, [19] report a median duration of hospital stay of 12.7 day. Based on these estimates, it likely takes a policy approximately four to six weeks to influence the COVID-19 fatality growth rate.

Table 2 shows the results from restricting restaurant, bar, gym, spa, retail and movie theater operations for the baseline and low population databases. Some partial closures appear to be at least as effective as full shutdowns, especially in the bar and restaurant sector. Among them, letting restaurants and bars open at 25% of capacity appears to be the most effective. Baseline estimates imply that, counties imposing this limit combination see 6-week ahead fatality growth that is 3.37% lower in level than a county which keeps both open without any restrictions. This magnitude is meaningful. During the sample period, counties saw a mean growth rate in the weekly fatalities of 7.84%. The 3.37% figure implies a 43% reduction from the mean rate of 7.84%. By comparison, the estimates imply that locales which completely closed both restaurants and bars saw a subsequent fatality growth reduction of only 16%. As shown in the S3 Table, this difference is statistically significant. Note that, although the estimated coefficient on the *Bars Closed*, *Restaurants 25%* combination is even larger than that on *Bars 25%*, *Restaurants 25%* we cannot draw strong conclusions because the pre-trend analysis in the S4 Table shows significant pre-trends that indicate declining fatality growth near *Bars Closed*, *Restaurants 25%* policy introduction. The other two bar and restaurant combinations that produce consistently negative and statistically significant estimates across forecast horizons and databases are restricting bars to outside service while allowing restaurants to open to 50% of capacity and closing bars while allowing restaurants open to 50% of capacity. (The estimated coefficients on both these policies are significantly smaller than what we observe for full closures. See the S3 Table.) The one policy that appears to be counterproductive is letting bars open to 25% of capacity and restaurants to 50%. However, based on the pre-trends analysis in the S4 Table, this policy combination also tends to follow large growth in rate of COVID-19 fatalities, which violates one of the conditions necessary for us to interpret the estimates.

**Table 2:** Baseline and low population counties forecast regressions 4 and 6 weeks ahead.

	Restaurant, Bar, Gym, Spa, Retail and Movie Theater Estimates							
	Baseline Data				Low Population Counties			
VARIABLES	Mean_t+4_	S.E.	Mean_t+6_	S.E.	Growth_t+4_	S.E.	Growth_t+6_	S.E.
Bars Closed, Rest Closed	-1.141**	0.499	-1.265***	0.464	-1.229**	0.560	-1.202**	0.523
Bars Closed, Rest Out	-0.453	0.468	-1.660***	0.424	-0.590	0.540	-2.009***	0.480
Bars Out, Rest Out	0.445	0.514	-0.699	0.460	0.693	0.592	-0.615	0.527
Bars Closed, Rest 25%	-0.498	0.525	-0.243	0.531	-0.931	0.602	-0.714	0.607
Bars Out, Rest 25%	-4.294***	0.932	-3.500***	0.963	-6.280***	1.241	-4.304**	1.691
Bars 25%, Rest 25%	-2.896***	0.505	-3.379***	0.500	-3.233***	0.619	-3.736***	0.617
Bars Closed, Rest 50%	-0.850**	0.342	-1.115***	0.329	-0.988**	0.392	-1.198***	0.371
Bars Out, Rest 50%	1.204*	0.651	-0.521	0.703	1.067	0.740	-0.745	0.790
Bars 25%, Rest 50%	-0.620	0.501	0.222	0.502	-0.717	0.554	0.142	0.550
Bars 50%, Rest 50%	-0.538**	0.263	-0.364	0.242	-0.751**	0.305	-0.534*	0.276
Bars Closed, Rest >50%	1.715***	0.470	-0.389	0.357	1.809***	0.510	-0.363	0.390
Bars 25%, Rest >50%	4.155***	0.768	2.362***	0.646	4.439***	0.857	2.465***	0.718
Bars 50%, Rest >50%	0.034	0.293	-0.123	0.278	-0.175	0.322	-0.343	0.305
Gyms Closed	-0.714*	0.408	-1.144***	0.389	-0.737	0.462	-1.091**	0.440
Gyms 25%	0.550	0.369	0.685*	0.363	0.873**	0.416	1.151***	0.407
Gyms 50%	0.244	0.281	-0.266	0.272	0.451	0.312	-0.083	0.299
Spas Closed	2.656***	0.409	2.678***	0.412	2.723***	0.458	2.755***	0.462
Spas 25%	1.247***	0.407	0.799**	0.399	0.882*	0.458	0.389	0.443
Spas 50%	1.186***	0.261	1.601***	0.259	1.099***	0.280	1.516***	0.278
Retail Closed	-0.461	0.534	-1.226**	0.534	-0.841	0.598	-1.880***	0.607
Retail 25%	-0.651**	0.263	-0.274	0.253	-0.477	0.298	-0.092	0.284
Retail 50%	-0.722***	0.216	-0.614***	0.200	-0.638***	0.246	-0.449**	0.226
Movies Closed	0.323	0.378	-0.132	0.353	0.289	0.423	-0.337	0.394
Movies 25%	1.166***	0.312	0.559*	0.291	1.150***	0.341	0.436	0.315
Movies 50%	0.883***	0.276	0.310	0.265	0.833***	0.299	0.131	0.284
Observations	66,321		66,321		58,860		58,860	
Adjusted R-squared	0.0844		0.0873		0.0815		0.0843	
Control	YES		YES		YES		YES	

The table shows results of estimating Eq (1), where the dependent variable is the *j* week ahead (from date *t*) fatality growth. Each explanatory variable is a dummy variable equal to 1 if that policy is in place on date *t* and 0 otherwise. Capacity limits over 50% (including full openings) are the omitted policies. Lagged fatality growth, current and lagged cumulative fatalities per capita, demographic and weather controls are all included in the regressions, but estimated coefficients are not reported in the table. *Baseline Data* estimates include all counties. The *Low Population* sample excludes the five most populous counties in each state. Standard errors are clustered at the county level and are robust to heteroskedasticity. Significance Key

* 10%

** 5%

*** 1%.

The retail sector is another area where partial closures are associated with lower COVID-19 fatality growth rates than full lockdowns. While closing them appears ineffective, introducing 50% capacity limits future fatalities. Indeed, a 50% capacity limit appears to limit future fatality growth more than even a 25% limit does. It may be that policy makers had some idea that this might be true. S1 Table indicates about 30% of all the county weeks saw capacity restricted to 50% while only 7% and 12% include a total closure or a 25% capacity limit respectively.

For gyms, Table 2 indicates that full closures reduced the COVID-19 fatality growth rate. Both the 4 and 6-week forecast horizons in the baseline and low population data produce negative and statistically significant estimates. Importantly, the pre-trend analysis in the S4 Table shows that fatalities were increasing prior to the introduction of gym restrictions. This implies that the reduced fatality growth rate estimates from gym closures are not simply reflecting an existing trend. Other gym constraints, however, appear to be either ineffective or even counterproductive. Based on the estimates, one would conclude that gyms should either be closed altogether or allowed to reopen to over 50% of capacity. Unlike restaurant, bar and gym restrictions, the results in Table 2 imply that all restrictions on spas and movie theaters are either ineffective or counterproductive. Spa closures appear particularly counterproductive. However, policies were not as harmful as the coefficients in Table 2 imply because spa closures were introduced when fatality growth rates were abnormally high.

The S5–S7 Tables show a variety of robustness checks. In the S5 Table, we remove all lagged dependent variables from the specification. The estimated coefficients on the policy variables are similar to those shown in Table 2, suggesting that including controls for recent fatalities does not bias our main findings. In the S6 Table, we winsorize the fatality growth variable to ensure that results are not driven by outliers. Again, the findings are similar to what we observe in Table 2. The S7 Table shows results when we include county fixed effects. This specification absorbs some important variation in the data since it does not allow us to exploit cross-county variation for areas where policies are in effect for most of the sample period. Still, we continue to find that restricting bars and restaurants to 25% capacity is even more effective than closing them completely. We also continue to find that many restrictions are ineffective or counterproductive.

Table 3 shows results for the near-neighbor samples. Overall, the estimates reinforce the earlier findings. In Table 4, we summarize all of the findings in Tables 2 and 3 and we also compare them to the pre-trends analysis. We base our main conclusions on the “Overall” column, where we indicate a (-) or (+) if two conditions are met: (1) the Tables 2 and 3 columns show that the policy is helpful (-) or harmful (+); and (2) the pre-trends analysis indicates no trend or a trend of the opposite sign. In support of the earlier discussion, several combinations of partial capacity restrictions for bars and restaurants are as effective as full shutdowns. For gyms, while full closures are followed by a lower COVID-19 fatality growth rate, partial closures may be counterproductive relative to leaving capacity unrestricted. For spas and movie theaters, we find that constraints are either ineffective or counterproductive.

**Table 3:** Near neighbor regressions.

	100 Mile Radius				200 Mile Radius			
VARIABLES	Growth_t+4_	S.E.	Growth_t+6_	S.E.	Growth_t+4_	S.E.	Growth_t+6_	S.E.
Bars Closed, Rest Closed	-0.782	0.710	-0.903	0.606	-2.022**	0.992	-2.603***	0.817
Bars Closed, Rest Out	-0.793	0.681	-1.452**	0.626	1.554	1.035	1.640*	0.918
Bars Out, Rest Out	1.142	0.763	-0.371	0.685	1.676*	1.013	-0.461	0.850
Bars Closed, Rest 25%	-0.178	0.832	0.296	0.895	0.726	1.049	1.075	0.897
Bars Out, Rest 25%	-4.969***	1.313	-4.306***	1.378	-3.066**	1.374	-3.530***	1.266
Bars 25%, Rest 25%	-2.549***	0.788	-3.319***	0.720	-4.152***	0.816	-5.123***	0.777
Bars Closed, Rest 50%	-1.011**	0.460	-1.059**	0.432	-0.260	0.582	-0.783	0.527
Bars Out, Rest 50%	0.233	0.769	-1.945**	0.800	0.335	0.796	-2.160***	0.672
Bars 25%, Rest 50%	-0.552	0.652	-0.260	0.621	-0.343	0.761	-0.128	0.744
Bars 50%, Rest 50%	-0.699*	0.399	-0.354	0.360	-1.233***	0.474	-1.112***	0.431
Bars Closed, Rest >50%	2.871***	0.734	0.384	0.513	1.455	1.007	0.958	0.735
Bars 25%, Rest >50%	3.849***	0.951	1.628**	0.721	1.454	0.919	0.235	0.773
Bars 50%, Rest >50%	-0.507	0.418	-0.298	0.380	-1.468**	0.577	-1.072**	0.509
Gyms Closed	-0.984*	0.554	-1.112**	0.525	0.007	0.731	0.140	0.649
Gyms 25%	0.837	0.523	0.966**	0.490	1.934**	0.881	1.974**	0.783
Gyms 50%	0.511	0.392	-0.108	0.375	0.778	0.519	0.155	0.484
Spas Closed	2.162***	0.599	2.408***	0.594	2.039**	0.862	2.495***	0.844
Spas 25%	1.277**	0.590	0.777	0.561	-0.899	0.923	-1.677**	0.848
Spas 50%	0.921**	0.367	1.575***	0.356	0.654	0.522	1.460***	0.500
Retail Closed	-0.675	0.770	-0.893	0.799	2.843**	1.293	1.794*	1.026
Retail 25%	-0.557	0.378	-0.215	0.364	-1.162**	0.508	-0.637	0.508
Retail 50%	-0.388	0.313	-0.469*	0.285	-0.651*	0.369	-0.565*	0.338
Movies Closed	0.014	0.558	-0.812	0.515	-1.401**	0.679	-2.011***	0.636
Movies 25%	0.655	0.446	0.074	0.417	0.490	0.576	-0.331	0.528
Movies 50%	0.127	0.394	-0.143	0.372	-1.093**	0.496	-1.081**	0.467
Observations	37,298		37,298		23,336		23,336	
Adjusted R-squared	0.0892		0.0938		0.0930		0.0990	
Control	YES		YES		YES		YES	
Near Neighbor Policy	YES		YES		YES		YES	

The regressions in this table repeat those in Table 2 with the exception that near neighbor policies are included as control variables. For inclusion in the sample, a county must not lie on its state’s border. In addition, there must be a matching non-border county in another state with a population centroid within X miles of the target county (where X is 100 or 200 miles). Among the set of possible matches, the one closest in a multi-dimensional hedonic distance is selected based on equation (333) in S1 Methods. Standard errors are clustered at the county level and are robust to heteroskedasticity. Significance Key

* 10%

** 5%

*** 1%.

**Table 4:** Summary of coefficients.

	Pre-trend	Table 2	Table 3	Overall
Bars Closed, Rest Closed	+	−	−	**−**
Bars Closed, Rest Out	+*	−		
Bars Out, Rest Out	+			
Bars Closed, Rest 25%				
Bars Out, Rest 25%	−	−	−	
Bars 25%, Rest 25%	+*	−	−	**−**
Bars Closed, Rest 50%	+	−	−	**−**
Bars Out, Rest 50%	+	−	−	**−**
Bars 25%, Rest 50%	−*			
Bars 50%, Rest 50%	−*	−	−	
Bars Closed, Rest >50%	−	+		
Bars 25%, Rest >50%	+*	+	+	
Bars 50%, Rest >50%	−*		−	
Gyms Closed	+*	−	−	**−**
Gyms 25%	+*	+	+	
Gyms 50%	−*			
Spas Closed	+*	+	+	
Spas 25%	+*	+		
Spas 50%	−	+	+	**+**
Retail Closed	−*	−	+	
Retail 25%	−			
Retail 50%	+*	−	−	**−**
Movies Closed	+*		−	
Movies 25%		+	+	**+**
Movies 50%		+	−	

This table summarizes the relationship between the findings in Tables 2 and 3 and fatality pre-trends. Full results from the pre-trend analysis are in the S3 Table. For the pre-trends analysis, “−” and “+” indicate negative and positive estimated coefficients (respectively) with at least one statistically significant at the 10% level and no significant coefficients of the opposite sign. A * indicates that at least three of the estimates are statistically significant and there are no significant coefficients of the opposite sign. For the Tables 2 and 3 analyses “−” and “+” indicate negative and positive estimated coefficients for the 4 and 6 horizons (respectively) with at least two statistically significant at the 10% level and no significant estimates of the opposite sign. The “Overall” column includes an icon if two conditions are met: (1) the Tables 2 and 3 columns have the same icon and (2) the pre-trends analysis must indicate no trend or one of the opposite sign.

## Conclusions

Government entities initially responded to the COVID-19 pandemic with policies that shut down an array of businesses. The literature indicates that some of these policies likely helped curb future fatalities. A natural question is whether it is possible to achieve similar results with less stringent policies. This paper uses data on partial openings of restaurants, bars, gyms, spas, retail establishments, and movie theaters to shed light on that question.

For gyms, the evidence indicates that shutting them down completely is beneficial, but other restrictions are ineffective. Counties that did not limit gym capacity below 50% saw no faster growth in COVID-19 fatality growth than those that did; unless they closed them entirely. When it comes to movie theaters, spas and other personal care services, all restrictions appear to be unhelpful or even counterproductive.

The fact that no county has placed tighter restrictions on restaurants than bars means that we must consider policy pairs for these establishments. We analyze the effectiveness of policies that restrict bars, given a particular restriction on restaurants. As one might expect, we find that closing them completely is then followed by a reduction in COVID-19 fatality growth. However, less restrictive pairs that limit bars to outdoor service generate similar results. For example, limiting bars and restaurants to 25% of capacity is followed by greater estimated reduction in COVID-19 fatality growth than full closures. Closing bars or restricting them to outdoor service while letting restaurants open to 50% of capacity generally also reduces fatality growth, though somewhat less than closing them completely. Why these pairs seem particularly effective at slowing deaths due to COVID-19 is a question we leave open to future research.

It may seem surprising that some capacity restrictions fail to reduce fatality growth due to COVID-19, or that some partial restrictions do more to reduce it than full shutdowns. However, these findings may reflect unintended consequences, where rules meant to reduce risks have the opposite effect. Although difficult to test without detailed location data, it is possible that people respond by substituting into riskier activities. The fact that these types of patterns have been documented in other safety settings [20–22] suggests that substitution effects could be at work. A definitive answer will, of course, require additional data and analysis.

Whenever one undertakes analysis of policy effectiveness, potential endogeneity issues arise. For example, [23] report large voluntary reductions in economic activity at the beginning of the pandemic. Our tests include a number of control variables and filters, as well as the near-neighbor analysis, to mitigate these concerns. Nevertheless, no set of controls or tests can fully eliminate the chance that regression results are coincidental (i.e. due to endogeneity) rather than causal. In the end, we can state whether fatality growth rose or fell after initiating various policies while controlling for a wide range of factors that might support alternative hypotheses. The results suggest that, while a subset of the policies put into place worked, others were ineffective.

This paper focuses on one particular benefit of business restrictions: slowing the rate of growth in COVID-19 fatalities. Future researchers might want to examine the costs. Clearly, higher capacity constraints do less economic and social damage than lower constraints. But, just how large of a difference is an issue that future articles should address. On the economic front, closures and restrictions lead to business bankruptcies and those often lead their owners and employees into financial distress. Beyond these economic costs there are social ones as well. To what degree did partial restrictions help reduce social problems like family breakups, drug addiction and other issues tied to financial hardship and social isolation. Before we can fully assess the value of full or even partial capacity restrictions we will need answers to all of these questions.

We acknowledge that there are some are natural limitations in applying the results of this study to policymaking. For example, we cannot be sure that our results apply outside of the United States or to communities with high vaccination rates (because our sample ends in December 2020). However, given the reduction in economic activity that shutdowns cause, one clear policy implication is that businesses should never fully shut down if partial opening does at least as well. The evidence in this paper shows that, in many cases, full shutdowns are unnecessary. Selective use of restrictions based on evidence regarding their efficacy might help policymakers gain trust from the public that some interventions can be helpful. This is especially important given the wide dispersion in public trust during the pandemic.[24] Of course, vaccines, therapeutics, and the virus itself may change over time. In devising policy responses based on the estimates produced here, policy makers should also pay close attention to the impact of these types of developments.

## Materials and Methods

The methods section is organized as follows. The “Data” section describes the datasets that we use in the analysis, the construction of the weekly COVID-19 fatality growth rates that we use in the regressions, and filters that we impose. The “Empirical Model” section describes the basic regression model that we use to estimate the relationship between fatality growth due to COVID-19 and the policy variables for the full dataset, as well as low population counties. The “Near-Neighbors” section describes the method used to identify counties for the near-neighbor regressions.

### Data

Daily fatalities by county are from USAFacts.org. We aggregate these data to create a Wednesday-to-Wednesday series of weekly fatality growth, by county. We convert the weekly fatality counts to weekly growth rates, written as

$$G_{i,t}=\ln(\frac{D_{i,t}}{D_{i,t-1}})$$ (2)

where *G*_*i*,*t*_ is the natural log of the growth in COVID-19 deaths in county *i* in week *t* and *D*_*i*,*t*_ equals the total deaths during the week. The variable *G*_*i*,*t*_ is the dependent variable in all regressions. The S2 Table Panel B summarizes the fatality growth variable, *G*_*i*,*t*._

#### Demographics

County demographics are from the most recently available data from the U.S. Census. We use: the fraction of the population that identifies itself as Black, Hispanic, Asian, Native American and Other than White; age-related variables that control for the fraction of the population over 65 years, the fraction over 85 years; the fraction of total population residing in nursing homes; and housing and population density, expressed in units per square mile. We also collect county per capita income, as reported by the Bureau of Economic Analysis. Since physical health is correlated with severe disease, we collect information on the fraction of the population that smokes, is obese, or has diabetes from the County Health Rankings organization.

#### Weather

Local weather conditions may influence COVID-19’s spread [25–27]. We obtain weather data for every weather station in the National Climatic Data Center for 2020. We use reports from the three stations closest to the county’s population centroid and average them to produce estimates for temperature, dew point and rainfall. We include five weather related controls: average temperature; hot and humid weekdays; hot and humid weekends; cold weekdays; and cold weekends. Weekdays and weekends are separated to allow for the possibility that weekday weather impacts behavior differently from weekend weather. A day is considered hot and humid if the average temperature exceeds 80 degrees and the dew point exceeds 60 degrees Fahrenheit. A day is considered as cold if the temperature is below 60 degrees. Using these measures, weekdays have between 0 and 5 hot and humid or cold days. Weekends have 0, 1 or 2 such days.

#### Policies

We hand-collect collect policy data on a range of actions taken by all U.S. state and county governments. These are the same data in [8], but we supplement them to account for partial openings of restaurants, bars, gyms, spas, retail establishments, and movie theaters. We introduce five categories of restrictions for restaurants and bars: completely closed, outdoor dining only, indoor up to 25% capacity, indoor greater than 25% up to 50% capacity, and indoor over 50% of capacity. Gyms, spas, retail establishments, and movie theaters have four categories: closed, indoor up to 25% capacity, indoor up to 50% capacity and indoor over 50% capacity. In some cases, governments use population limits (e.g. no more than 50 people indoors at a time) or limits on the number people per square feet. When this was the case, we tried to convert the restrictions into capacity percentages by combining data on average facility size and capacity limits. The restrictions on bars and restaurants present a unique correlation structure. No county ever restricted restaurant capacity more than bars. Thus, all of the tests focus on the impact of a restriction on bar capacity, given a particular restriction on restaurant capacity.

#### Filters

The total database has 178,467 observations and covers the period March 1, 2020 to December 31, 2020. Since prior rates of growth in COVID-19 fatalities are likely predictive of the current rate, we include six lags of weekly fatality growth in all of our regressions. That requires us to drop all date-county observations until six weeks after a county records its first fatality. Although this reduces the database to 67,535 observations (the Baseline Data”), it guarantees that tests of whether a policy alters the trajectory of deaths due to COVID-19 are conducted on areas where the virus is actually present.

A second filter, used for only a subset of the analyses, produces the “low population” sample. In it, we drops the five most populous counties in each state from the sample. We call this the Low Population dataset, which has 45,824 observations. This sample is of particular interest because, although all of our analyses focus on policies at the county level, many restrictions come from State Governors’ orders. In these cases, elected officials are likely to focus their policy efforts based on concerns about their state’s more populated areas. From the perspective of our tests, removing the state’s most populous counties increases the likelihood that, if a policy’s enactment is then followed by reduced fatality growth in the low population dataset, the reduction is due to the policy rather than politicians reacting to future forecasts.

#### Data Availability

Sample data and information on how to purchase a license for the full dataset are available at: https://som.yale.edu/covid-restrictions.

### Empirical model

The basic regression model forecasts the *t*+*k* period rate of fatality growth based on data as of period *t*:

$$100G_{c,}{}_{t+k}=\alpha +\sum_{p}\beta_{p,k}D_{c,p,t}+controls$$

In addition to the county demographic, income, and weather variables described above, the following fatality and time controls: six lags of weekly fatality growth; total deaths to date; the time since the county’s first reported death; and the number of days since March 1, 2020. Lagged growth controls for serial correlation in the fatality rate. Total deaths to date indicate population’s likely level of immunity from those that were infected but survived. We also interact this measure with the lagged growth rates, in case the level of fatalities itself influences the degree which past fatality growth predicts future fatality growth. The days since the first county fatality controls for the total time the virus has been circulating. Finally, the days since March 1, 2020 allows for advancements in medical care that might lower the rate of growth in the number of COVID-19 deaths.

### Near-neighbors

The near-neighbors analysis focuses on a set of matched counties that are in different states and that lie near (but not on) state borders. One county acts as the treatment area (with the policy) and the other as a control. We follow [8] and introduce samples that only include counties that are nearby, but do not lie along a state’s border. We refer to these as interior counties. For an interior county to enter the database, its population centroid must lie within 100 or 200 miles (depending on the version of the filter imposed) of a given interior county’s population centroid. If there are multiple possible matches, we select the paring that is closest in characteristic space, based on a Euclidean measure. We refer to these as the Neighbor 100 and Neighbor 200 miles samples. These samples have 24,550 and 38,728 observations with average distances between county centroids of 85 and 127 miles respectively.

The distance function that we use to select among possible pairs is

$$d_{ij}=\sqrt{{\sum_{k=1}^{n}\left(\frac{h_{ki}-h_{kj}}{\sigma_{k}}\right)}^{2}}$$ (3)

Where the *h*_*ki*_ represent county *i*’s hedonic measure *k* and σ_*k*_ is the standard deviation of the hedonic measure across counties. This implies that a one standard deviation difference in hedonic *k* between the target county *i* and another county *j* is coded as one. The hedonics used in (333) are: per capita income, the fraction of the population over age 85, population density, housing density, weekly temperature, and rain. Matching on distance, along with demographics and weather should produce county pairs with similar infection transmission rates.

## Supporting information

S1 Methods The methods section is organized as follows. The “Data” section describes the datasets that we use in the analysis, the construction of the weekly COVID-19 fatality growth rates that we use in the regressions, and filters that we impose. Data access information can be found in S1 Methods at the end of this section. The “Empirical Model” section describes the basic regression model that we use to estimate the relationship between fatality growth due to COVID-19 and the policy variables for the full dataset, as well as low population counties. The “Near-Neighbors” section describes the method used to identify counties for the near-neighbor regressions.
(PDF)

S1 Table Summary statistics. This table shows the number of observations for the restrictions on restaurants, bars, gyms, spas, retail establishments, and movie theaters that we analyze in this paper. Variable names indicate the business type and the associated capacity limit, with “>50%” indicating a capacity limit over 50% (including 100% or full capacity). Total includes the entire database, before imposing filters. Baseline Data is all available county data beginning 6 weeks after the first recorded fatality. Low Population is the baseline data after the 5 most populous counties in each state have been dropped. Neighbor 100 is the baseline data using only counties that are not on the state border and for which a matching non-border county within 100 in another state exists. Neighbor 200 is the same as Neighbor 100, but the matching distance is extended to 200 miles. The percentages in each cell indicate the percent of observations in the sample where we observe the policy.
(PDF)

S2 Table Distributions of policy duration and fatality growth rate. Panel A displays the number of weeks restrictions are left in place across dates and counties in the Baseline database. Column headers indicate percentiles. Panel B displays the distribution for the growth in the fatality rate for the four samples that we analyze. The fatality growth rate is the dependent variable in all regressions.
(PDF)

S3 Table Coefficient significance tests against bars and restaurants closed. This table reports F-tests for the hypothesis that the coefficient values reported in the Baseline Data regressions in Table 2 equal the coefficient values for the full closure policy Bars Closed, Restaurants Closed. The values in the table are p-values. *** indicates significance at the 1% level; ** indicates significance at 5% = **; * indicates significance at 10%.
(PDF)

S4 Table Pre-trends analysis: Residual fatality growth near policy introductions. This table calculates residuals from a regression of week-ahead change in deaths (Growth(t+1)) during weeks t+j, where j = -2 through +2 relative to the introduction of policy i. Control variables are: current cumulative deaths in the county, lagged changes in deaths per capita, time controls, weather information, and demographic data are included in the regression. We also include all policies that are already in place as of period t from Table 2 other than the newly implemented policy i, where policy i is the policy listed in the first column. Meant+i denotes the week t+j average change fatality growth times 100. *** denotes significance at the 1% level; ** denotes 5% significance; * denotes 10% significance.
(PDF)

S5 Table Baseline forecast regressions 4 and 6 weeks ahead, without the lagged dependent variable. The table shows results of estimating Eq (1), where the dependent variable is the j week ahead (from date t) fatality growth. The data and specification are identical to the regressions using the Baseline Data in Table 2 in the main paper, except remove the lagged fatality growth and lagged cumulative fatalities per capita variables from the specification. Each explanatory variable is a dummy variable equal to 1 if that policy is in place on date t and 0 otherwise. Capacity limits over 50% (including full openings) are the omitted policies. Demographic and weather controls are all included in the regressions, but estimated coefficients are not reported in the table. The Baseline Data include all counties. Standard errors are clustered at the county level. Significance Key: * 10%; ** 5%; *** 1%.
(PDF)

S6 Table Baseline forecast regressions 4 and 6 weeks ahead using Winsorized Data. The table shows results of estimating Eq (1), where the dependent variable is the j week ahead (from date t) fatality growth. The data and specification are identical to the regressions using the Baseline Data in Table 2 in the main paper, except we winsorize the fatality growth variable at 99% and 1%. Each explanatory variable is a dummy variable equal to 1 if that policy is in place on date t and 0 otherwise. Capacity limits over 50% (including full openings) are the omitted policies. Lagged fatality growth, current and lagged cumulative fatalities per capita, demographic and weather controls are all included in the regressions, but estimated coefficients are not reported in the table. The Baseline Data include all counties. Standard errors are clustered at the county level. Significance Key: * 10%; ** 5%; *** 1%.
(PDF)

S7 Table Baseline forecast regressions 4 and 6 weeks ahead, with fixed effects. The table shows results of estimating Eq (1), where the dependent variable is the j week ahead (from date t) fatality growth. The data and specification are identical to the regressions using the Baseline Data in Table 2 in the main paper, except we substitute week and county fixed effects for the time-invariant controls. Each explanatory variable is a dummy variable equal to 1 if that policy is in place on date t and 0 otherwise. Capacity limits over 50% (including full openings) are the omitted policies. Lagged fatality growth, current and lagged cumulative fatalities per capita, county fixed effects, and week fixed effects are all included in the regressions, but estimated coefficients are not reported in the table. The Baseline Data include all counties. Standard errors are clustered at the county level. Significance Key: * 10%; ** 5%; *** 1%.
(PDF)

## References

[1] Banholzer N, van Weenen E, Lison A, Cenedese A, Seeliger A, Kratzwald B, et al. Estimating the effects of non-pharmaceutical interventions on the number of new infections with COVID-19 during the first epidemic wave. PLoS ONE. 2021;16: e0252827. doi: 10.1371/journal.pone.0252827 34077448 PMC8171941

[2] Brauner JM, Mindermann S, Sharma M, Johnston D, Salvatier J, Gavenčiak T, et al. Inferring the effectiveness of government interventions against COVID-19. Science. 2021 Feb 19;371(6531):eabd9338. doi: 10.1126/science.abd9338 Epub 2020 Dec 15. ; PMCID: PMC7877495. 33323424 PMC7877495

[3] Chang S, Pierson E, Koh PW, Gerardin J, Redbird B, Grusky D, et al. Mobility network models of COVID-19 explain inequities and inform reopening. Nature. 2021 Jan;589(7840):82–87. doi: 10.1038/s41586-020-2923-3 Epub 2020 Nov 10. . 33171481

[4] Chapman S. Indiana plan to catch up on statewide testing, WTHR. 2020 May 1 [Cited 2021 Oct 23]. Available from: https://www.wthr.com/article/news/investigations/13-investigates/indiana-plan-catch-statewide-testing/531-6f5a24f6-5155-46cf-98d5-c273761cc0f8.

[5] Chaudhry R, Dranitsaris G, Mubashir T, Bartoszko J, Riazi S. A country level analysis measuring the impact of government actions, country preparedness and socioeconomic factors on COVID-19 mortality and related health outcomes. EClinicalMedicine. 2020 Aug;25:100464. doi: 10.1016/j.eclinm.2020.100464 32838237 PMC7372278

[6] Holtz D, Zhaoa M, Benzellb SG, Caoa CY, Rahimiana MA, Yanga J, et al. Interdependence and the cost of uncoordinated responses to COVID-19, Proceedings of the National Academy of Sciences of the United States of America. 2020 August 18:117. 19837–19843, doi: 10.1073/pnas.2009522117 32732433 PMC7443871

[7] Ling D, Wang C, Zhou T. A first look at the impact of COVID-19 on commercial real estate prices: Asset-level evidence. Review of Asset Pricing Studies. 2020;10:669–704.

[8] Spiegel M, Tookes H. Business restrictions and COVID-19 Fatalities. Review of Financial Studies. 2021;34:5266–5308.

[9] Valencia M, Becerra JE, Reyes JC, Castro KG (2020) Assessment of early mitigation measures against COVID-19 in Puerto Rico: March 15-May 15, 2020. PLoS ONE 15(10): e0240013. doi: 10.1371/journal.pone.0240013 33052958 PMC7556465

[10] Abouk R, Heydari B. The immediate effect of COVID-19 policies on social distancing behavior in the United States. Public Health Reports. 2021;136: 245–52. doi: 10.1177/0033354920976575 33400622 PMC8093844

[11] Askitas N, Tatsiramos K, Verheyden B. Lockdown strategies, mobility patterns and COVID-19. COVID. Economics. 2020;23: 263–302.

[12] Bongaerts D, Mazzola F, Wagner W. Closed for business: The mortality impact of business closures during the Covid-19 pandemic. PLoS ONE 2021 May 14;16(5): e0251373. doi: 10.1371/journal.pone.0251373 33989322 PMC8121299

[13] Di Domenico L, Pullano G, Sabbatini CE, Boëlle PY, Colizza V. Modelling safe protocols for reopening schools during the COVID-19 pandemic in France. Nat Commun. 2021 Feb 16;12(1):1073. doi: 10.1038/s41467-021-21249-6 33594076 PMC7887250

[14] Courtemanche C, Garuccio J, Le A, Pinkston J, Yelowitz A. Strong Social Distancing Measures In The United States Reduced The COVID-19 Growth Rate. Health Aff (Millwood). 2020 Jul;39(7):1237–1246. doi: 10.1377/hlthaff.2020.00608 32407171

[15] Centers for Disease Control and Prevention. Interim Clinical Guidance for Management of Patients with Confirmed Coronavirus Disease (COVID-19). 2021 Feb 16 [Cited 2021 Oct 23]. Available From: https://www.cdc.gov/coronavirus/2019-ncov/hcp/clinical-guidance-management-patients.html.

[16] Kissane E, Daily COVID-19 Data is About to Get Weird. The COVID Tracking Project. 2020 Nov 24 [Cited 2021 Oct 23]. Available From: https://covidtracking.com/blog/daily-covid-19-data-is-about-to-get-weird.

[17] DeGregory P. ‘Unfair’ indoor dining rules send Queens patrons to Nassau: lawsuit. 2020 August 31 [Cited 2021 Oct 23]. Available From: https://nypost.com/2020/08/31/indoor-dining-rules-send-queens-patrons-over-to-nassau-lawsuit/. doi: 10.1177/1078155219873014 31500517

[18] Pomranz M. States with open restaurants are (predictably) becoming tourist hot spots. 2020 May 20 [Cited 2021 Oct 23]. Available From: https://www.foodandwine.com/news/open-restaurants-attract-tourists-wisconsin.

[19] Lewnard JA, Liu VX, Jackson ML, Schmidt MA, Jewell BL, Flores JP, et al. Incidence, clinical outcomes, and transmission dynamics of severe coronavirus disease 2019 in California and Washington: prospective cohort study. BMJ. 2020 May 22;369:m1923. doi: 10.1136/bmj.m1923 32444358 PMC7243800

[20] Jones T, Tomcheck P. Pedestrian accidents in marked and unmarked crosswalks: A quantitative study. ITE Journal, Institute of Transportation Engineers. 2000;70: 42–46.

[21] Risa AE. Adverse incentives from improved technology: traffic safety regulation in Norway. Southern Economic Journal. 1994;60: 844–857.

[22] Schneider DK, Grandhi RK, Bansal P, Kuntz GE 4th, Webster KE, Logan K, et al. Current state of concussion prevention strategies: a systematic review and meta-analysis of prospective, controlled studies. Br J Sports Med. 2017 Oct;51(20):1473–1482. doi: 10.1136/bjsports-2015-095645 27251896

[23] Goolsbee A, Syverson C. Fear, lockdown, and diversion: Comparing drivers of pandemic economic decline 2020. Journal of Public Economics. 2021;193: 104311. doi: 10.1016/j.jpubeco.2020.104311 33262548 PMC7687454

[24] Gozgor G. Global evidence on the determinants of public trust in governments during the COVID-19. Appl Res Qual Life. 2021 Feb;5:1–20. doi: 10.1007/s11482-020-09902-6 33564341 PMC7862976

[25] Baker RE, Yang W, Vecchi GA, Metcalf CJE, Grenfell BT. Susceptible supply limits the role of climate in the early SARS-CoV-2 pandemic. Science. 2020 Jul 17;369(6501):315–319. doi: 10.1126/science.abc2535 32423996 PMC7243362

[26] Carlson CJ, Gomez ACR, Bansal S, Ryan SJ. Misconceptions about weather and seasonality must not misguide COVID-19 response. Nat Commun. 2020 Aug 27;11(1):4312. doi: 10.1038/s41467-020-18150-z 32855406 PMC7452887

[27] Guo C, Bo Y, Lin C, Li HB, Zeng Y, Zhang Y, Hossain MS, Chan JWM, Yeung DW, Kwok KO, Wong SYS, Lau AKH, Lao XQ. Meteorological factors and COVID-19 incidence in 190 countries: An observational study. Sci Total Environ. 2021 Feb 25;757:143783. doi: 10.1016/j.scitotenv.2020.143783 33257056 PMC7682932

