# Supplementary material for: All or nothing? Partial business shutdowns and COVID-19 fatality growth
Source: PLoS One. 2022 Feb 9;17(2):e0262925. doi: 10.1371/journal.pone.0262925 (PMC8827474; doi:10.1371/journal.pone.0262925)
Supplement: S1 Methods — The “Data” section describes the datasets that we use in the analysis, the construction of the weekly COVID-19 fatality growth rates that we use in the regressions, and filters that we impose. Data access information can be found in S1 Methods at the end of this section. The “Empirical Model” section describes the basic regression model that we use to estimate the relationship between fatality growth due to COVID-19 and the policy variables for the full dataset, as well as low population counties. The “Near-Neighbors” section describes the method used to identify counties for the near-neighbor regressions. (PDF) [file pone.0262925.s001.pdf]

S1 Methods.

The methods section is organized as follows. The "Data" section describes the datasets that we use in the analysis, the construction of the weekly COVID-19 fatality growth rates that we use in the regressions, and filters that we impose. Data access information can be found in S1 Methods at the end of this section. The "Empirical Model" section describes the basic regression model that we use to estimate the relationship between fatality growth due to COVID-19 and the policy variables for the full dataset, as well as low population counties. The "Near-Neighbors" section describes the method used to identify counties for the near-neighbor regressions.

Data

Daily fatalities by county are from USAFacts.org. We aggregate these data to create a Wednesday-to-Wednesday series of weekly fatality growth, by county. We convert the weekly fatality counts to weekly growth rates, written as

$$G_{i,t} = \ln\left(\frac{D_{i,t}}{D_{i,t-1}}\right) \quad (1)$$

where $G_{i,t}$ is the natural log of the growth in COVID-19 deaths in county *i* in week *t* and $D_{i,t}$ equals the total deaths during the week. The variable $G_{i,t}$ is the dependent variable in all regressions. The S2 Table Panel B summarizes the fatality growth variable, $G_{i,t}$.

Demographics. County demographics are from the most recently available data from the U.S. Census. We use: the fraction of the population that identifies itself as Black, Hispanic, Asian, Native American and Other than White; age-related variables that control for the fraction of the population over 65 years, the fraction over 85 years; the fraction of total population residing in nursing homes; and housing

and population density, expressed in units per square mile. We also collect county per capita income, as reported by the Bureau of Economic Analysis. Since physical health is correlated with severe disease, we collect information on the fraction of the population that smokes, is obese, or has diabetes from the County Health Rankings organization.

Weather. Local weather conditions may influence COVID-19's spread [1-3]. We obtain weather data for every weather station in the National Climatic Data Center for 2020. We use reports from the three stations closest to the county's population centroid and average them to produce estimates for temperature, dew point and rainfall. We include five weather related controls: average temperature; hot and humid weekdays; hot and humid weekends; cold weekdays; and cold weekends. Weekdays and weekends are separated to allow for the possibility that weekday weather impacts behavior differently from weekend weather. A day is considered hot and humid if the average temperature exceeds 80 degrees and the dew point exceeds 60 degrees Fahrenheit. A day is considered as cold if the temperature is below 60 degrees. Using these measures, weekdays have between 0 and 5 hot and humid or cold days. Weekends have 0, 1 or 2 such days.

Policies. We hand-collect collect policy data on a range of actions taken by all U.S. state and county governments. These are the same data in [4], but we supplement them to account for partial openings of restaurants, bars, gyms, spas, retail establishments, and movie theaters. We introduce five categories of restrictions for restaurants and bars: completely closed, outdoor dining only, indoor up to 25% capacity, indoor greater than 25% up to 50% capacity, and indoor over 50% of capacity. Gyms, spas, retail establishments, and movie theaters have four categories: closed, indoor up to 25% capacity, indoor up to 50% capacity and indoor over 50% capacity. In some cases, governments use population limits (e.g. no more than 50 people indoors at a time) or limits on the number people per square feet. When this was the case, we tried to convert the restrictions into capacity percentages by combining data on average facility size and capacity limits. The restrictions on bars and restaurants present a

unique correlation structure. No county ever restricted restaurant capacity more than bars. Thus, all of the tests focus on the impact of a restriction on bar capacity, given a particular restriction on restaurant capacity.

Filters. The total database has 178,467 observations and covers the period March 1, 2020 to December 31, 2020. Since prior rates of growth in COVID-19 fatalities are likely predictive of the current rate, we include six lags of weekly fatality growth in all of our regressions. That requires us to drop all date-county observations until six weeks after a county records its first fatality. Although this reduces the database to 67,535 observations (the Baseline Data"), it guarantees that tests of whether a policy alters the trajectory of deaths due to COVID-19 are conducted on areas where the virus is actually present.

A second filter, used for only a subset of the analyses, produces the "low population" sample. In it, we drops the five most populous counties in each state from the sample. We call this the Low Population dataset, which has 45,824 observations. This sample is of particular interest because, although all of our analyses focus on policies at the county level, many restrictions come from State Governors' orders. In these cases, elected officials are likely to focus their policy efforts based on concerns about their state's more populated areas. From the perspective of our tests, removing the state's most populous counties increases the likelihood that, if a policy's enactment is then followed by reduced fatality growth in the low population dataset, the reduction is due to the policy rather than politicians reacting to future forecasts.

Data Availability. Sample data and information on how to purchase a license for the full dataset are available at: https://som.yale.edu/covid-restrictions.

Empirical Model

The basic regression model forecasts the *t+k* period rate of fatality growth based on data as of period *t*:

$$d_{ij} = \sqrt{\sum_{k=1}^{n} \left(\frac{h_{ki} - h_{kj}}{\sigma_k} \right)^2} \quad (2)$$

In addition to the county demographic, income, and weather variables described above, the following fatality and time controls: six lags of weekly fatality growth; total deaths to date; the time since the county's first reported death; and the number of days since March 1, 2020. Lagged growth controls for serial correlation in the fatality rate. Total deaths to date indicate population's likely level of immunity from those that were infected but survived. We also interact this measure with the lagged growth rates, in case the level of fatalities itself influences the degree which past fatality growth predicts future fatality growth. The days since the first county fatality controls for the total time the virus has been circulating. Finally, the days since March 1, 2020 allows for advancements in medical care that might lower the rate of growth in the number of COVID-19 deaths.

Near-Neighbors

The near-neighbors analysis focuses on a set of matched counties that are in different states and that lie near (but not on) state borders. One county acts as the treatment area (with the policy) and the other as a control. We follow [4] and introduce samples that only include counties that are nearby, but do not lie along a state's border. We refer to these as interior counties. For an interior county to enter the database, its population centroid must lie within 100 or 200 miles (depending on the version of the filter imposed) of a given interior county's population centroid. If there are multiple possible matches, we select the paring that is closest in characteristic space, based on a Euclidean measure. We refer to these as the Neighbor 100 and Neighbor 200 miles samples. These samples have 24,550 and 38,728 observations with average distances between county centroids of 85 and 127 miles respectively.

The distance function that we use to select among possible pairs is

$$d_{ij} = \sqrt{\sum_{k=1}^{n} \left(\frac{h_{ki} - h_{kj}}{\sigma_k} \right)^2} \quad (3)$$

Where the h_{ki} represent county *i*'s hedonic measure *k* and σ_k is the standard deviation of the hedonic measure across counties. This implies that a one standard deviation difference in hedonic *k* between the target county *i* and another county *j* is coded as one. The hedonics used in (3) are: per capita income, the fraction of the population over age 85, population density, housing density, weekly temperature, and rain. Matching on distance, along with demographics and weather should produce county pairs with similar infection transmission rates.

References

1. Baker RE, Yang W, Vecchi GA, Metcalf CJE, Grenfell BT. Susceptible supply limits the role of climate in the early SARS-CoV-2 pandemic. Science. 2020 Jul 17;369(6501):315-319. doi: 10.1126/science.abc2535.
2. Carlson CJ, Gomez ACR, Bansal S, Ryan SJ. Misconceptions about weather and seasonality must not misguide COVID-19 response. Nat Commun. 2020 Aug 27;11(1):4312. doi: 10.1038/s41467-020-18150-z.
3. Guo C, Bo Y, Lin C, Li HB, Zeng Y, Zhang Y, Hossain MS, Chan JWM, Yeung DW, Kwok KO, Wong SYS, Lau AKH, Lao XQ. Meteorological factors and COVID-19 incidence in 190 countries: An observational study. Sci Total Environ. 2021 Feb 25;757:143783. doi: 10.1016/j.scitotenv.2020.143783.
4. Spiegel M, Tookes H. Business restrictions and COVID-19 Fatalities. Review of Financial Studies. 2021;34:5266-5308.
