# Supplementary material for: All or nothing? Partial business shutdowns and COVID-19 fatality growth
Source: PLoS One. 2022 Feb 9;17(2):e0262925. doi: 10.1371/journal.pone.0262925 (PMC8827474; doi:10.1371/journal.pone.0262925)
Supplement: S1 Table — This table shows the number of observations for the restrictions on restaurants, bars, gyms, spas, retail establishments, and movie theaters that we analyze in this paper. Variable names indicate the business type and the associated capacity limit, with “>50%” indicating a capacity limit over 50% (including 100% or full capacity). Total includes the entire database, before imposing filters. Baseline Data is all available county data beginning 6 weeks after the first recorded fatality. Low Population is the baseline data after the 5 most populous counties in each state have been dropped. Neighbor 100 is the baseline data using only counties that are not on the state border and for which a matching non-border county within 100 in another state exists. Neighbor 200 is the same as Neighbor 100, but the matching distance is extended to 200 miles. The percentages in each cell indicate the percent of observations in the sample where we observe the policy. (PDF) [file pone.0262925.s002.pdf]

S1 Table. Summary Statistics.

Variable	Total	Baseline Data	Low Population	Neighbor 100	Neighbor 200
Bars Closed, Rest Closed	24,417<br>10.58%	2,349<br>3.54%	1,899<br>3.23%	1,263<br>3.39%	618<br>2.65%
Bars Closed, Rest Out	5,852<br>2.54%	1,929<br>2.91%	1,610<br>2.74%	1,030<br>2.76%	186<br>0.80%
Bars Out, Rest Out	3,346<br>1.45%	1,658<br>2.50%	1,436<br>2.44%	886<br>2.38%	690<br>2.96%
Bars Closed, Rest 25%	3,097<br>1.34%	1,188<br>1.79%	979<br>1.66%	512<br>1.37%	159<br>0.68%
Bars Out, Rest 25%	431<br>0.19%	264<br>0.40%	110<br>0.19%	135<br>0.36%	60<br>0.26%
Bars 25%, Rest 25%	2,495<br>1.08%	1,238<br>1.87%	953<br>1.62%	609<br>1.63%	324<br>1.39%
Bars Closed, Rest 50%	14,084<br>6.10%	7,514<br>11.33%	6,531<br>11.10%	4,270<br>11.45%	1,949<br>8.35%
Bars Out, Rest 50%	2,694<br>1.17%	771<br>1.16%	676<br>1.15%	480<br>1.29%	304<br>1.30%
Bars 25%, Rest 50%	5,395<br>2.34%	2,654<br>4.00%	2,478<br>4.21%	1,530<br>4.10%	1,063<br>4.56%
Bars 50%, Rest 50%	41,451<br>17.96%	16,259<br>24.52%	14,230<br>24.18%	8,995<br>24.12%	6,313<br>27.05%
Bars Closed, Rest >50%	6,489<br>2.81%	2,553<br>3.85%	2,409<br>4.09%	1,181<br>3.17%	421<br>1.80%
Bars Out, Rest >50%	48<br>0.02%	8<br>0.01%	8<br>0.01%	4<br>0.01%	4<br>0.02%
Bars 25%, Rest >50%	473<br>0.20%	346<br>0.52%	305<br>0.52%	262<br>0.70%	163<br>0.70%
Bars 50%, Rest >50%	20,280<br>8.79%	8,195<br>12.36%	7,799<br>13.25%	5,016<br>13.45%	3,744<br>16.04%
Bars >50%, Rest >50%	96,407<br>41.77%	18,866<br>28.45%	17,139<br>29.12%	10,858<br>29.11%	7,198<br>30.85%
Gyms Closed	31,468<br>13.63%	6,327<br>9.54%	5,287<br>8.98%	3,595<br>9.64%	1,860<br>7.97%
Gyms 25%	24,582<br>10.65%	8,352<br>12.59%	6,862<br>11.66%	4,613<br>12.37%	1,800<br>7.71%
Gyms 50%	52,147<br>22.59%	24,394<br>36.78%	21,351<br>36.27%	13,162<br>35.29%	9,196<br>39.41%
Gyms >50%	120,856<br>52.36%	27,388<br>41.30%	25,491<br>43.31%	16,011<br>42.93%	10,518<br>45.07%

Spas Closed	26,738<br>11.58%	3,490<br>5.26%	2,840<br>4.83%	1,681<br>4.51%	878<br>3.76%
Spas 25%	14,891<br>6.45%	5,494<br>8.28%	4,494<br>7.64%	2,718<br>7.29%	1,137<br>4.87%
Spas 50%	61,428<br>26.61%	28,567<br>43.07%	25,199<br>42.81%	15,700<br>42.09%	8,811<br>37.76%
Spas >50%	126,102<br>54.63%	28,835<br>43.48%	26,388<br>44.83%	17,237<br>46.21%	12,521<br>53.66%
Retail Closed	17,146<br>7.43%	1,679<br>2.53%	1,225<br>2.08%	746<br>2.00%	169<br>0.72%
Retail 25%	28,802<br>12.48%	10,032<br>15.13%	8,611<br>14.63%	5,716<br>15.33%	2,635<br>11.29%
Retail 50%	71,153<br>30.83%	30,871<br>46.55%	27,450<br>46.64%	17,923<br>48.05%	12,303<br>52.72%
Retail >50%	111,976<br>48.51%	23,882<br>36.01%	21,706<br>36.88%	12,995<br>34.84%	8,268<br>35.43%
Movies Closed	40,659<br>17.62%	10,986<br>16.56%	9,290<br>15.78%	6,096<br>16.34%	3,119<br>13.37%
Movies 25%	35,555<br>15.40%	10,896<br>16.43%	9,275<br>15.76%	5,792<br>15.53%	3,381<br>14.49%
Movies 50%	77,775<br>33.70%	32,848<br>49.53%	29,778<br>50.59%	19,485<br>52.24%	13,764<br>58.98%
Movies >50%	75,327<br>32.64%	11,823<br>17.83%	10,730<br>18.23%	6,056<br>16.24%	3,168<br>13.58%
N	230,812	66,321	58,860	37,298	23,336

This table shows the number of observations for the restrictions on restaurants, bars, gyms, spas, retail establishments, and movie theaters that we analyze in this paper. Variable names indicate the business type and the associated capacity limit, with “>50%” indicating a capacity limit over 50% (including 100% or full capacity). *Total* includes the entire database, before imposing filters. *Baseline Data* is all available county data beginning 6 weeks after the first recorded fatality. *Low Population* is the baseline data after the 5 most populous counties in each state have been dropped. *Neighbor 100* is the baseline data using only counties that are not on the state border and for which a matching non-border county within 100 in another state exists. *Neighbor 200* is the same as *Neighbor 100*, but the matching distance is extended to 200 miles. The percentages in each cell indicate the percent of observations in the sample where we observe the policy.
