# Supplementary material for: All or nothing? Partial business shutdowns and COVID-19 fatality growth
Source: PLoS One. 2022 Feb 9;17(2):e0262925. doi: 10.1371/journal.pone.0262925 (PMC8827474; doi:10.1371/journal.pone.0262925)
Supplement: S2 Table — Panel A displays the number of weeks restrictions are left in place across dates and counties in the Baseline database. Column headers indicate percentiles. Panel B displays the distribution for the growth in the fatality rate for the four samples that we analyze. The fatality growth rate is the dependent variable in all regressions. (PDF) [file pone.0262925.s003.pdf]

S2 Table. Distributions of Policy Duration and Fatality Growth Rate.

	5%	25%	Median	75%	95%	Mean
Panel A: Policy Duration						
Bars Closed, Rest Closed	2.714	5.571	9.000	11.000	12.286	8.148
Bars Closed, Rest Out	0.286	0.857	2.857	6.000	11.993	4.025
Bars Closed, Rest 25%	0.571	1.000	2.000	4.857	17.143	4.110
Bars Closed, Rest 50%	0.143	0.714	3.571	11.429	19.714	6.179
Bars Closed, Rest >50%	0.286	0.857	3.429	15.714	26.714	7.791
Bars Out, Rest Out	0.571	1.143	2.571	4.000	9.857	3.753
Bars Out, Rest 25%	0.143	0.250	0.571	6.321	17.714	4.452
Bars Out, Rest 50%	0.286	0.571	0.714	5.000	5.286	2.361
Bars Out, Rest >50%	0.657	1.000	1.000	1.857	3.571	1.686
Bars 25%, Rest 50%	0.714	0.714	3.000	9.571	15.314	4.971
Bars 25%, Rest 25%	0.714	0.714	2.143	12.714	15.286	6.595
Bars 25%, Rest >50%	0.714	0.714	0.714	7.000	9.714	3.311
Bars 50%, Rest 50%	0.286	2.286	5.714	18.714	30.571	10.769
Bars 50%, Rest >50%	0.286	1.429	7.143	15.143	30.286	10.117
Bars >50%, Rest >50%	19.229	27.143	54.571	57.429	66.571	45.406
Gyms Closed	2.857	5.714	8.571	13.857	24.429	10.695
Gyms 25%	0.714	3.000	10.857	23.429	34.286	13.603
Gyms 50%	2.143	14.000	18.143	25.857	32.286	18.100
Gyms >50%	14.857	27.143	52.000	61.429	69.429	45.368
Spas Closed	3.386	8.286	10.571	13.143	27.143	11.714
Spas 25%	0.714	5.714	6.143	27.286	35.286	13.130
Spas 50%	4.314	16.000	27.714	31.714	32.571	23.666
Spas >50%	15.429	27.429	57.429	65.429	71.429	52.119
Retail Closed	6.286	8.286	9.714	10.857	40.000	12.471
Retail 25%	2.000	5.000	17.286	33.286	39.000	19.319
Retail 50%	1.714	5.000	18.357	32.000	34.714	18.611
Retail >50%	3.729	21.429	56.286	61.143	86.857	45.928
Movies Closed	1.000	5.714	10.571	14.000	40.000	12.768
Movies 25%	1.286	3.429	5.429	23.429	31.714	12.395
Movies 50%	1.000	5.000	19.000	29.000	32.286	17.373
Movies >50%	1.714	9.143	27.143	56.571	66.000	30.544
Panel B: Fatality Growth Rate in %						
Baseline Data	0.000	0.000	2.097	9.531	33.647	7.844
Low Population	0.000	0.000	1.563	9.820	36.772	7.997
Neighbor 100	0.000	0.000	1.942	9.309	33.647	7.564
Neighbor 200	0.000	0.000	2.151	9.798	34.831	7.946

Panel A displays the number of weeks restrictions are left in place across dates and counties in the Baseline database. Column headers indicate percentiles. Panel B displays the distribution for the growth in the fatality rate for the four samples that we analyze. The fatality growth rate is the dependent variable in all regressions.
