# Supplementary material for: All or nothing? Partial business shutdowns and COVID-19 fatality growth
Source: PLoS One. 2022 Feb 9;17(2):e0262925. doi: 10.1371/journal.pone.0262925 (PMC8827474; doi:10.1371/journal.pone.0262925)
Supplement: S3 Table — This table reports F-tests for the hypothesis that the coefficient values reported in the Baseline Data regressions in Table 2 equal the coefficient values for the full closure policy Bars Closed, Restaurants Closed. The values in the table are p-values. *** indicates significance at the 1% level; ** indicates significance at 5% = **; * indicates significance at 10%. (PDF) [file pone.0262925.s004.pdf]

S3 Table. Coefficient Significance Tests Against Bars and Restaurants Closed.

	Baseline Data	
	t+4	t+6
Bars Closed, Rest Out	0.394	0.603
Bars Out, Rest Out	0.004***	0.135
Bars Closed, Rest 25%	0.686	0.174
Bars Out, Rest 25%	0.166	0.293
Bars 25%, Rest 25%	0.083*	0.036**
Bars Closed, Rest 50%	0.092*	0.046**
Bars Out, Rest 50%	0.000***	0.015**
Bars 25%, Rest 50%	0.218	0.005***
Bars 50%, Rest 50%	0.468	0.046**
Bars Closed, Rest >50%	0.001***	0.541
Bars 25%, Rest >50%	0.000***	0.024**
Bars 50%, Rest >50%	0.665	0.203

This table reports F-tests for the hypothesis that the coefficient values reported in the Baseline Data regressions in Table 2 equal the coefficient values for the full closure policy *Bars Closed, Restaurants Closed*. The values in the table are p-values. *** indicates significance at the 1% level; ** indicates significance at 5% = **; * indicates significance at 10%.
