# Supplementary material for: All or nothing? Partial business shutdowns and COVID-19 fatality growth
Source: PLoS One. 2022 Feb 9;17(2):e0262925. doi: 10.1371/journal.pone.0262925 (PMC8827474; doi:10.1371/journal.pone.0262925)
Supplement: S4 Table — This table calculates residuals from a regression of week-ahead change in deaths (Growth(t+1)) during weeks t+j, where j = -2 through +2 relative to the introduction of policy i. Control variables are: current cumulative deaths in the county, lagged changes in deaths per capita, time controls, weather information, and demographic data are included in the regression. We also include all policies that are already in place as of period t from Table 2 other than the newly implemented policy i, where policy i is the policy listed in the first column. Meant+i denotes the week t+j average change fatality growth times 100. *** denotes significance at the 1% level; ** denotes 5% significance; * denotes 10% significance. (PDF) [file pone.0262925.s005.pdf]

S4 Table. Pre-trends analysis: Residual Fatality Growth near Policy Introductions.

	Week *t-2*		Week *t-1*		Week *t*		Week *t+1*		Week *t+2*	
	Mean	*p*-value	Mean	*p*-value	Mean	*p*-value	Mean	*p*-value	Mean	*p*-value
Bars Closed, Rest Close	-0.689	1.016	2.081**	1.013	1.118	0.944	0.564	0.802	0.588	0.924
Bars Closed, Rest Out	2.716***	0.899	3.959***	0.946	2.204***	0.694	2.839***	0.738	1.471**	0.597
Bars Out, Rest Out	1.156	0.941	1.808**	0.759	2.946***	0.800	0.719	0.628	0.202	0.536
Bars Closed, Rest 25%	0.741	1.341	-1.964**	0.801	0.052	0.857	2.697**	1.256	-0.172	0.708
Bars Out, Rest 25%	-2.043**	0.928	-0.349	1.950	3.635	3.096	-2.684*	1.348	-1.994	1.294
Bars 25%, Rest 25%	1.203	1.043	-0.670	0.845	2.191**	0.921	3.589***	1.034	4.479***	1.000
Bars Closed, Rest 50%	-0.448	0.573	-0.213	0.442	-0.500	0.466	0.509	0.507	1.489***	0.488
Bars Out, Rest 50%	0.774	0.742	1.808***	0.688	0.983	0.751	-0.724	0.608	-0.796	0.537
Bars 25%, Rest 50%	-1.192**	0.504	0.119	0.638	-1.748***	0.453	-1.518***	0.584	-1.046*	0.560
Bars 50%, Rest 50%	-0.565	0.498	-1.326***	0.370	-1.239***	0.380	-0.720*	0.372	-0.585	0.376
Bars Closed, Rest >50%	-2.226***	0.525	-1.699***	0.563	-1.116**	0.560	-0.577	0.657	0.349	0.838
Bars 25%, Rest >50%	2.232*	1.235	-0.044	1.023	4.546***	1.243	5.681***	1.313	8.379***	1.509
Bars 50%, Rest >50%	-0.698	0.494	-1.602***	0.435	-0.967**	0.445	-0.654	0.507	-1.964***	0.412
Gyms Closed	2.426***	0.878	3.486***	0.834	3.848***	0.828	3.636***	0.766	3.687***	0.791
Gyms 25%	1.222*	0.724	0.653	0.671	1.251**	0.626	2.740***	0.746	2.369***	0.667
Gyms 50%	-0.428	0.393	0.013	0.392	-0.898**	0.393	-1.724***	0.303	-0.934**	0.368
Spas Closed	1.173	1.666	4.367***	1.583	5.547***	1.528	5.162***	1.556	3.947**	1.746
Spas 25%	3.336***	0.870	2.618***	0.793	4.098***	0.786	4.702***	0.865	5.346***	0.851
Spas 50%	-0.380	0.479	-0.494	0.474	-1.587***	0.476	-0.840*	0.434	0.203	0.490
Retail Closed	-2.560*	1.270	4.161	3.911	-3.006*	1.694	-0.988	1.722	-5.175***	1.381

Retail 25%	-1.601*	0.856	-0.750	0.901	-1.230*	0.673	-0.075	0.828	0.225	0.977
Retail 50%	1.435***	0.522	3.229***	0.543	2.066***	0.481	0.279	0.432	-0.565	0.371
Movies Closed	1.315**	0.599	1.908***	0.576	1.348**	0.525	1.323***	0.461	1.138**	0.452
Movies 25%	-0.439	0.631	0.758	0.696	-1.315***	0.430	1.555**	0.664	1.538**	0.687
Movies 50%	0.373	0.355	0.663**	0.323	-0.752***	0.270	-1.109**	0.455	0.373	0.513

This table calculates residuals from a regression of week-ahead change in deaths (Growth(*t+1*)) during weeks *t+j*, where *j* = -2 through +2 relative to the introduction of policy *i*. Control variables are: current cumulative deaths in the county, lagged changes in deaths per capita, time controls, weather information, and demographic data are included in the regression. We also include all policies that are already in place as of period *t* from Table 2 other than the newly implemented policy i, where policy i is the policy listed in the first column. $Mean_{t+i}$ denotes the week *t+j* average change fatality growth times 100. *** denotes significance at the 1% level; ** denotes 5% significance; * denotes 10% significance.
