# Supplementary material for: All or nothing? Partial business shutdowns and COVID-19 fatality growth
Source: PLoS One. 2022 Feb 9;17(2):e0262925. doi: 10.1371/journal.pone.0262925 (PMC8827474; doi:10.1371/journal.pone.0262925)
Supplement: S5 Table — The table shows results of estimating Eq (1), where the dependent variable is the j week ahead (from date t) fatality growth. The data and specification are identical to the regressions using the Baseline Data in Table 2 in the main paper, except remove the lagged fatality growth and lagged cumulative fatalities per capita variables from the specification. Each explanatory variable is a dummy variable equal to 1 if that policy is in place on date t and 0 otherwise. Capacity limits over 50% (including full openings) are the omitted policies. Demographic and weather controls are all included in the regressions, but estimated coefficients are not reported in the table. The Baseline Data include all counties. Standard errors are clustered at the county level. Significance Key: * 10%; ** 5%; *** 1%. (PDF) [file pone.0262925.s006.pdf]

S5 Table. Baseline Forecast Regressions 4 and 6 Weeks Ahead, without the Lagged Dependent Variable.

VARIABLES	$Mean_{t+4}$	S.E.	$Mean_{t+6}$	S.E.
Bars Closed, Rest Closed	-1.108**	0.503	-1.234***	0.464
Bars Closed, Rest Out	-0.406	0.468	-1.709***	0.418
Bars Out, Rest Out	0.401	0.515	-0.793*	0.454
Bars Closed, Rest 25%	-0.598	0.526	-0.330	0.526
Bars Out, Rest 25%	-4.291***	0.921	-3.386***	0.930
Bars 25%, Rest 25%	-2.939***	0.504	-3.389***	0.491
Bars Closed, Rest 50%	-0.848**	0.343	-1.130***	0.324
Bars Out, Rest 50%	1.145*	0.659	-0.678	0.703
Bars 25%, Rest 50%	-0.639	0.503	0.205	0.495
Bars 50%, Rest 50%	-0.495*	0.264	-0.274	0.238
Bars Closed, Rest >50%	1.716***	0.471	-0.375	0.357
Bars 25%, Rest >50%	4.206***	0.768	2.381***	0.636
Bars 50%, Rest >50%	0.059	0.294	-0.102	0.274
Gyms Closed	-0.738*	0.410	-1.223***	0.384
Gyms 25%	0.541	0.372	0.673*	0.359
Gyms 50%	0.247	0.282	-0.288	0.268
Spas Closed	2.506***	0.410	2.406***	0.406
Spas 25%	1.304***	0.406	0.798**	0.391
Spas 50%	1.200***	0.262	1.637***	0.254
Retail Closed	-0.494	0.533	-1.272**	0.520
Retail 25%	-0.619**	0.263	-0.205	0.248
Retail 50%	-0.721***	0.217	-0.621***	0.198
Movies Closed	0.265	0.379	-0.174	0.348
Movies 25%	1.154***	0.312	0.512*	0.286
Movies 50%	0.860***	0.278	0.305	0.261
Observations	66,321		66,321	
Adjusted R-squared	0.0823		0.0851	

The table shows results of estimating Equation (1), where the dependent variable is the *j* week ahead (from date *t*) fatality growth. The data and specification are identical to the regressions using the *Baseline Data* in Table 2 in the main paper, except remove the lagged fatality growth and lagged cumulative fatalities per capita variables from the specification. Each explanatory variable is a dummy variable equal to 1 if that policy is in place on date *t* and 0 otherwise. Capacity limits over 50% (including full openings) are the omitted policies. Demographic and weather controls are all included in the regressions, but estimated coefficients are not reported in the table. The *Baseline Data* include all counties. Standard errors are clustered at the county level. Significance Key: * 10%; ** 5%; *** 1%.
