# Supplementary material for: All or nothing? Partial business shutdowns and COVID-19 fatality growth
Source: PLoS One. 2022 Feb 9;17(2):e0262925. doi: 10.1371/journal.pone.0262925 (PMC8827474; doi:10.1371/journal.pone.0262925)
Supplement: S6 Table — The table shows results of estimating Eq (1), where the dependent variable is the j week ahead (from date t) fatality growth. The data and specification are identical to the regressions using the Baseline Data in Table 2 in the main paper, except we winsorize the fatality growth variable at 99% and 1%. Each explanatory variable is a dummy variable equal to 1 if that policy is in place on date t and 0 otherwise. Capacity limits over 50% (including full openings) are the omitted policies. Lagged fatality growth, current and lagged cumulative fatalities per capita, demographic and weather controls are all included in the regressions, but estimated coefficients are not reported in the table. The Baseline Data include all counties. Standard errors are clustered at the county level. Significance Key: * 10%; ** 5%; *** 1%. (PDF) [file pone.0262925.s007.pdf]

S6 Table. Baseline Forecast Regressions 4 and 6 Weeks Ahead Using Winsorized Data.

VARIABLES	$Mean_{t+4}$	S.E.	$Mean_{t+6}$	S.E.
Bars Closed, Rest Closed	-1.022**	0.467	-1.246***	0.438
Bars Closed, Rest Out	-0.407	0.438	-1.722***	0.396
Bars Out, Rest Out	0.557	0.482	-0.686	0.428
Bars Closed, Rest 25%	-0.379	0.502	-0.215	0.519
Bars Out, Rest 25%	-4.008***	0.878	-3.301***	0.923
Bars 25%, Rest 25%	-2.800***	0.468	-3.180***	0.479
Bars Closed, Rest 50%	-0.732**	0.316	-1.027***	0.303
Bars Out, Rest 50%	1.030*	0.555	-0.660	0.595
Bars 25%, Rest 50%	-0.604	0.461	0.129	0.454
Bars 50%, Rest 50%	-0.407*	0.244	-0.297	0.226
Bars Closed, Rest >50%	1.160***	0.387	-0.206	0.330
Bars 25%, Rest >50%	3.611***	0.625	2.375***	0.620
Bars 50%, Rest >50%	0.080	0.269	-0.134	0.253
Gyms Closed	-0.694*	0.378	-1.124***	0.361
Gyms 25%	0.634*	0.343	0.750**	0.336
Gyms 50%	0.172	0.257	-0.260	0.248
Spas Closed	2.549***	0.379	2.692***	0.384
Spas 25%	1.103***	0.373	0.802**	0.372
Spas 50%	1.173***	0.240	1.538***	0.235
Retail Closed	-0.573	0.514	-1.199**	0.518
Retail 25%	-0.559**	0.245	-0.246	0.236
Retail 50%	-0.790***	0.198	-0.632***	0.186
Movies Closed	0.265	0.349	-0.085	0.329
Movies 25%	1.048***	0.289	0.527**	0.268
Movies 50%	0.789***	0.253	0.348	0.242
Observations	66,321		66,321	
Adjusted R-squared	0.0928		0.0960	

The table shows results of estimating Equation (1), where the dependent variable is the *j* week ahead (from date *t*) fatality growth. The data and specification are identical to the regressions using the *Baseline Data* in Table 2 in the main paper, except we winsorize the fatality growth variable at 99% and 1%. Each explanatory variable is a dummy variable equal to 1 if that policy is in place on date *t* and 0 otherwise. Capacity limits over 50% (including full openings) are the omitted policies. Lagged fatality growth, current and lagged cumulative fatalities per capita, demographic and weather controls are all included in the regressions, but estimated coefficients are not reported in the table. The *Baseline Data* include all counties. Standard errors are clustered at the county level. Significance Key: * 10%; ** 5%; *** 1%.
