# Supplementary material for: All or nothing? Partial business shutdowns and COVID-19 fatality growth
Source: PLoS One. 2022 Feb 9;17(2):e0262925. doi: 10.1371/journal.pone.0262925 (PMC8827474; doi:10.1371/journal.pone.0262925)
Supplement: S7 Table — The table shows results of estimating Eq (1), where the dependent variable is the j week ahead (from date t) fatality growth. The data and specification are identical to the regressions using the Baseline Data in Table 2 in the main paper, except we substitute week and county fixed effects for the time-invariant controls. Each explanatory variable is a dummy variable equal to 1 if that policy is in place on date t and 0 otherwise. Capacity limits over 50% (including full openings) are the omitted policies. Lagged fatality growth, current and lagged cumulative fatalities per capita, county fixed effects, and week fixed effects are all included in the regressions, but estimated coefficients are not reported in the table. The Baseline Data include all counties. Standard errors are clustered at the county level. Significance Key: * 10%; ** 5%; *** 1%. (PDF) [file pone.0262925.s008.pdf]

**S7 Table. Baseline Forecast Regressions 4 and 6 Weeks Ahead, with Fixed Effects.**

| VARIABLES | $Mean_{t+4}$ | S.E. | $Mean_{t+6}$ | S.E. |
|---|---|---|---|---|
| Bars Closed, Rest Closed | -0.369 | 1.061 | -1.206 | 0.798 |
| Bars Closed, Rest Out | 0.148 | 1.001 | -0.925 | 0.854 |
| Bars Out, Rest Out | 1.476 | 1.028 | -0.289 | 0.914 |
| Bars Closed, Rest 25% | -0.076 | 1.059 | -0.247 | 0.940 |
| Bars Out, Rest 25% | -2.862 | 2.320 | -3.429 | 2.683 |
| Bars 25%, Rest 25% | -1.682 | 1.147 | -2.777*** | 0.957 |
| Bars Closed, Rest 50% | 0.618 | 0.719 | -0.102 | 0.973 |
| Bars Out, Rest 50% | 3.149*** | 0.907 | 0.641 | 0.884 |
| Bars 25%, Rest 50% | 0.614 | 1.010 | 1.024 | 1.015 |
| Bars 50%, Rest 50% | 0.036 | 0.754 | -0.149 | 0.724 |
| Bars Closed, Rest >50% | 2.369 | 2.314 | -1.614* | 0.905 |
| Bars 25%, Rest >50% | 3.778** | 1.616 | 0.784 | 1.085 |
| Bars 50%, Rest >50% | -0.089 | 0.695 | -0.421 | 0.591 |
| Gyms Closed | 0.334 | 0.790 | -0.196 | 0.631 |
| Gyms 25% | 0.912 | 0.722 | 1.499** | 0.599 |
| Gyms 50% | 0.517 | 0.782 | -0.202 | 0.680 |
| Spas Closed | 2.047*** | 0.768 | 1.660** | 0.744 |
| Spas 25% | -0.677 | 0.742 | -1.105 | 0.713 |
| Spas 50% | 0.460 | 0.626 | 1.115* | 0.620 |
| Retail Closed | 0.390 | 0.984 | 0.050 | 0.808 |
| Retail 25% | -1.148* | 0.661 | -1.444** | 0.608 |
| Retail 50% | -0.754 | 0.574 | -1.340*** | 0.463 |
| Movies Closed | 1.142** | 0.570 | 0.354 | 0.593 |
| Movies 25% | 1.257** | 0.628 | 0.690 | 0.744 |
| Movies 50% | 0.428 | 0.501 | -0.546 | 0.483 |
| Observations | 67634 | | 67634 | |
| Adjusted R-squared | 0.0798 | | 0.0732 | |

The table shows results of estimating Equation (1), where the dependent variable is the *j* week ahead (from date *t*) fatality growth. The data and specification are identical to the regressions using the *Baseline Data* in Table 2 in the main paper, except we substitute week and county fixed effects for the time-invariant controls. Each explanatory variable is a dummy variable equal to 1 if that policy is in place on date *t* and 0 otherwise. Capacity limits over 50% (including full openings) are the omitted policies. Lagged fatality growth, current and lagged cumulative fatalities per capita, county fixed effects, and week fixed effects are all included in the regressions, but estimated coefficients are not reported in the table. The *Baseline Data* include all counties. Standard errors are clustered at the county level. Significance Key: * 10%; ** 5%; *** 1%.
